# Network pharmacology combined with experimental verification to explore the potential mechanism of naringenin in the treatment of cervical cancer

**DOI:** 10.1038/s41598-024-52413-9

**Published:** 2024-01-22

**Authors:** Ji Zhou, Haoying Li, Ben Wu, Lemei Zhu, Qiao Huang, Zhenyu Guo, Qizhi He, Lin Wang, Xiaozhen Peng, Tianyao Guo

**Affiliations:** 1 Medical School, Changsha Social Work College, Changsha, China; 2 School of Public Health & Laboratory Medicine, Hunan University of Medicine, Huaihua, China; 3 The First Affiliated Hospital of Changsha Medical University, Changsha, China; 4 Wuzhou Medical college, Wuzhou, China; 5 Department of Pathology, The Second Xiangya Hospital of Central South University, Changsha, China; 6 Hunan Clinical Medical Research Center for Cancer Pathogenic Genes Testing and Diagnosis, The Second Xiangya Hospital of Central South University, Changsha, China

**Keywords:** Cancer, Computational biology and bioinformatics

## Abstract

Cervical cancer is the second leading cause of morbidity and mortality in women worldwide. Traditional treatment methods have become limited. Naringenin, a flavonoid abundant in various fruits and herbal medicines, has demonstrated anti-tumor properties among other effects. This research undertook to elucidate the mechanism of naringenin in the context of cervical cancer treatment by leveraging network pharmacology and performing experimental validation. Initial steps involved predicting potential naringenin targets and subsequently screening for overlaps between these targets and those related to cervical cancer, followed by analysis of their interrelationships. Molecular docking was subsequently utilized to verify the binding effect of the central target. Within the framework of network pharmacology, it was discovered that naringenin might possess anti-cancer properties specific to cervical cancer. Following this, the anti-tumor effects of naringenin on Hela cell viability, migration, and invasion were assessed employing CCK-8, transwell, wound healing assays, and western blotting. Experimental data indicated that naringenin attenuates the migration and invasion of Hela cells via downregulation EGFR/PI3K/AKT signaling pathway. Thus, our findings suggest that naringenin has therapeutic impacts on cervical cancer via multiple mechanisms, primarily by inhibiting the migration and invasion through the EGFR/PI3K/AKT/mTOR pathway. This study offers fresh insights for future clinical studies.

## Introduction

Cervical cancer (CC) stands as the second most prevalent gynecological malignancy affecting women worldwide^1,2^. Data from Global Oncology 2020 approximates 57,000 diagnoses and 37,000 fatalities per annum on a global scale. Predominantly, lower-income and lower-middle-income nations perpetually experience high incidences of cervical cancer, posing substantial threats to women’s health and longevity^3,4^. Contemporary treatment primarily relies on radiotherapy and chemotherapy, which indeed diminish a degree of mortality but cannot negate the considerable toxic and adverse effects emanating from prolonged chemotherapy^5,6^. In recent years, cervical cancer cells have developed resistance to chemotherapy drugs to curtail their efficacy. Additionally, novel immunotherapies such as adoptive cell therapy and antibody-targeted therapy fall short of expectations^7,8^. Consequently, the pressing demand for a new generation of efficacious, low-toxicity therapeutics for cervical cancer treatment remains unmet.

Traditional Chinese medicine (TCM) has always played an instrumental part in the treatment of diseases. Accumulating evidences illustrate that TCM’s active constituents display anti-tumor properties with minimal side effects^9,10^. Naringenin, a common dihydro-flavonoid active ingredient in fruits and traditional herbal medicines, exhibits extensive pharmacological potential such as anti-inflammatory, antioxidant, and anti-tumor activities^11,12^. Multiple researches have revealed naringenin is capable of hindering the proliferation of a wide spectrum of tumors, encompassing breast cancer, gastric cancer, liver cancer, and others^13–15^. For instance, naringenin can suppress cell migration via inflammatory and apoptotic pathways in breast cancer, and treat fatty liver disease induced by oxidative stress and fatty acid metabolism. One research demonstrated that naringenin can attenuate extra-cellular matrix levels, thereby inhibiting their growth^8^. Recent investigation even suggests a potential inhibitory effect of naringenin on COVID-19^16^. However, most extant studies pertaining to cervical cancer have been confined to single-agent studies, and the anti-cervical tumor mechanisms remain incompletely defined.

Network pharmacology, a novel technology underpinned by systems biology integrating bioinformatics and pharmacology, can systematically interpret and predict the interaction mechanisms of TCM and its compounds with a multitude of diseases via data analysis. The efficacy of network pharmacology and molecular docking in elucidating interactive mechanisms is well-documented^17–19^. In this study, we first constructed a “component-target-pathway” network using network pharmacology in conjunction with GEO datasets. Additionally, molecular docking was undertaken to ascertain potential interactions among the drugs with protein molecules. Concurrently, GO annotations and KEGG enrichment were utilized to examine the direct intersection targets, while PPI network analysis and Cytoscape were employed to pinpoint hub targets. Subsequent experiments aimed to validate the mechanism of naringenin in cervical cancer. Therefore, this study endeavours to predict and authenticate the target and plausible mechanism of naringenin in anti-cervical cancer via network pharmacology and cell-based experiments.

## Materials and methods

### Screening of potential targets

Naringenin structural formulas were searched in the PubChem and ChemSrc databases. The molecule structure was compared with relevant literature, and its CAS number and 2D structure map were used to draw a 3D structure map on ChemDraw, resulting in an exported sdf format file. The sdf file was uploaded to PharmMapper for target prediction, and the Uniprot ID obtained was converted to Geneofficial ID on the UniProt platform^20^.

### Construction of a cervical cancer-related targets database

Three series (GSE9750, GSE138080, GSE7803) were extracted from the NCBI GEO database using “Cervical Cancer” as a keyword. Among these expression datasets, GSE9750 includes 10 normal samples and 28 cervical cancer samples, GSE138080 includes 10 normal samples and 25 CC samples, and GSE7803 includes 10 normal samples and 28 CC samples. Differentially expressed genes were discerned employing a p < 0.05, and volcano and heatmap was plotted by https://www.bioinformatics.com.cn, an online platform for data analysis and visualization. Additional disease targets were unearthed through a GeneCards database search with “Cervical Cancer” as the keyword.

### Compounds-targets network and PPI network construction

After potential targets for naringenin and cervical cancer were identified, their intersection was found using Venny 2.1.0. These latent anti-cervical cancer targets of naringenin were imported into the STRING database for PPI analysis, using Homo sapiens as the species and a confidence score > 0.4 as filter parameters^21^. The PPI network map and target-pathway map were created using Cytoscape3.6.1 software. The hub targets were computed using the Degree algorithm in the CytoHubba plugin.

### Visual function enrichment analysis

The naringenin and cervical cancer targets were imported into Metascape for GO (Cellular Component, Molecular Function, Biological Process) and KEGG pathway enrichment analysis. The top 20 biological functions and pathways were selected according to gene enrichment and depicted in a histogram or bubble chart.

### Molecule docking identification

Molecular docking technology was harnessed to examine the binding effects of hub targets within the compound. The experimental procedure has previously been described^22^. After the 3D structure of naringenin was optimized, the corresponding 3D structure of proteins corresponding to the hub targets was procured from the PDB database. AutoDock Tools were used for hydrogenation, active pocket coordinates determination, and parameter setting. AutoDock Vina subsequently conducted molecular docking and recorded the binding energy.

### Cell culture and treatment

The HeLa cell line was obtained from the National Cell Bank of China (NCBC, Shanghai). The cells were cultivated in DMEM (Gibco, USA), supplemented with 10% FBS (Gibco, USA), 100 units/ml penicillin, and 100 mg/ml streptomycin (Beyotime, China). Culturing was conducted under standard conditions (5% CO_2_, 37 °C, 95% humidity), with cells consistently maintained in the growth phase. Naringenin (Macklin, China) was dissolved in DMSO (Gibco, USA) to create a 50 mM stock solution, which was then stored at − 20 °C. Treatments were administered to cells with and without naringenin at concentrations of 0 µM, 100 µM, and 200 µM.

### Cell viability assay

HeLa cells, representative of cervical cancer, were seeded into 96-well plates and exposed to varying concentrations of naringenin (0 μM, 100 μM, 200 μM) for 24 and 48 h at 37 °C. The medium was then replaced with the culture medium containing 10% CCK-8 (AbMole, USA) and incubated for an additional 2 h. The absorbance was measured at 490 nm using a microplate reader. All experiments were performed in triplicate.

### Wound healing assay

Following a previously described protocol^23^, wounds were created in the HeLa cell culture at 90% confluency using a 100 μl pipette tip. The cells were then cultured in DMEM supplemented with 10% FBS, in the presence or absence of naringenin at concentrations of 100 μM, 200 μM, and 0 μM for 24 h. Photomicrographs of each well were taken under a Leica DM2500 microscope (Germany).

### Cell invasion detection

Transwell chambers(Coring, USA) were utilized to detect cell invasion. Filters were coated with 50 mg/ml Matrigel (BD, USA) solution. HeLa cells were inoculated into the upper compartment of the transwell chambers (24 wells; 8 mm pore) and permitted to invade the lower chambers in a DMEM medium fortified with 20% FBS, in the presence or absence of naringenin for 24 h, the invading cells were fixed, stained, and quantified under a microscope.

### Western Bolt

Following the previously established procedure^24^, post 24 h naringenin treatment, the cells were lysed using RIPA buffer. Protein expressions of β-actin, MMP9, PI3K, AKT, and Caspase-3 were assessed through SDS-PAGE, transferred onto a PVDF membrane, the blots were cut prior to hybridization with antibodies, and cropped blots incubated overnight at 4 °C with primary antibodies including anti-MMP9 (Beyotime, 1:1000), anti-mTOR (Beyotime, 1:1000), anti-PI3K (Cell signaling, 1:1000), anti-p-PI3K (Cell signaling, 1:1000), anti-AKT (Cell signaling, 1:1000), anti-p-AKT (Cell signaling, 1:1000), anti-EGFR (ABclonal, 1:1000), anti-Caspase-3 (Beyotime, 1:1000), and anti-β-actin (Cell signaling, 1:1000). Secondary antibodies used were either GAM (goat anti-mouse IgG) or GAR (goat anti-rabbit IgG) (ABclonal, 1:2000).

### Statistical analysis

Data analysis was performed with GraphP ad Prism7.0 (La Jolla, CA, USA). Data were expressed as mean ± SD. P < 0.05 is considered as the significant difference.

## Result

### Analysis of predicted targets

**Figure 1:**
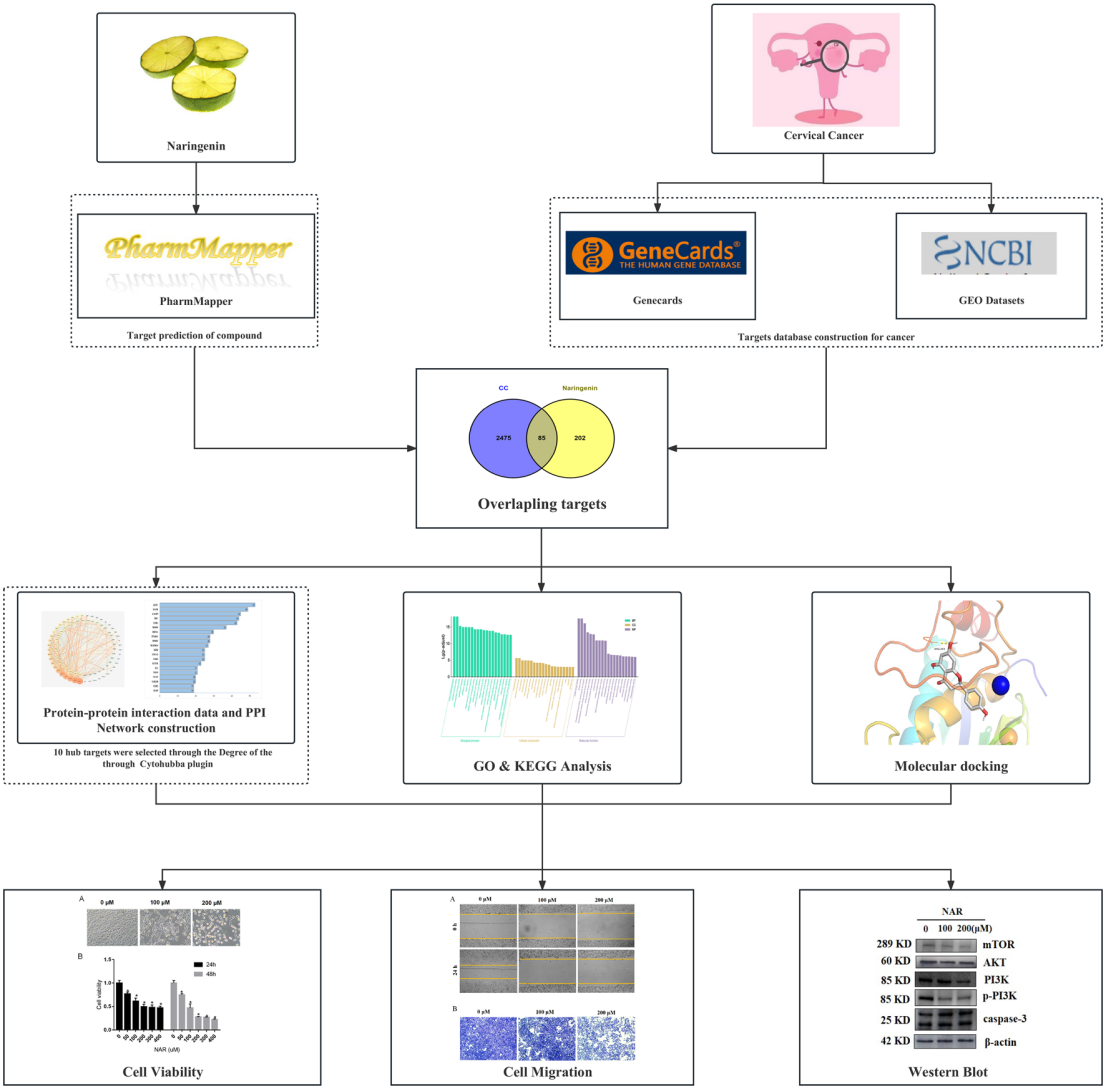
Flowchart of the research process.

Naringenin, an active compound in Chinese herbal medicine, is known for treating a variety of diseases. To elucidate the potential mechanism of naringenin in the treatment of cervical cancer, we devised a series of experiments for analysis (Fig. 1). We obtained 287 potential targets of naringenin that were predicted by Pharmmapper.

### Targets related to cervical cancer

**Figure 2:**
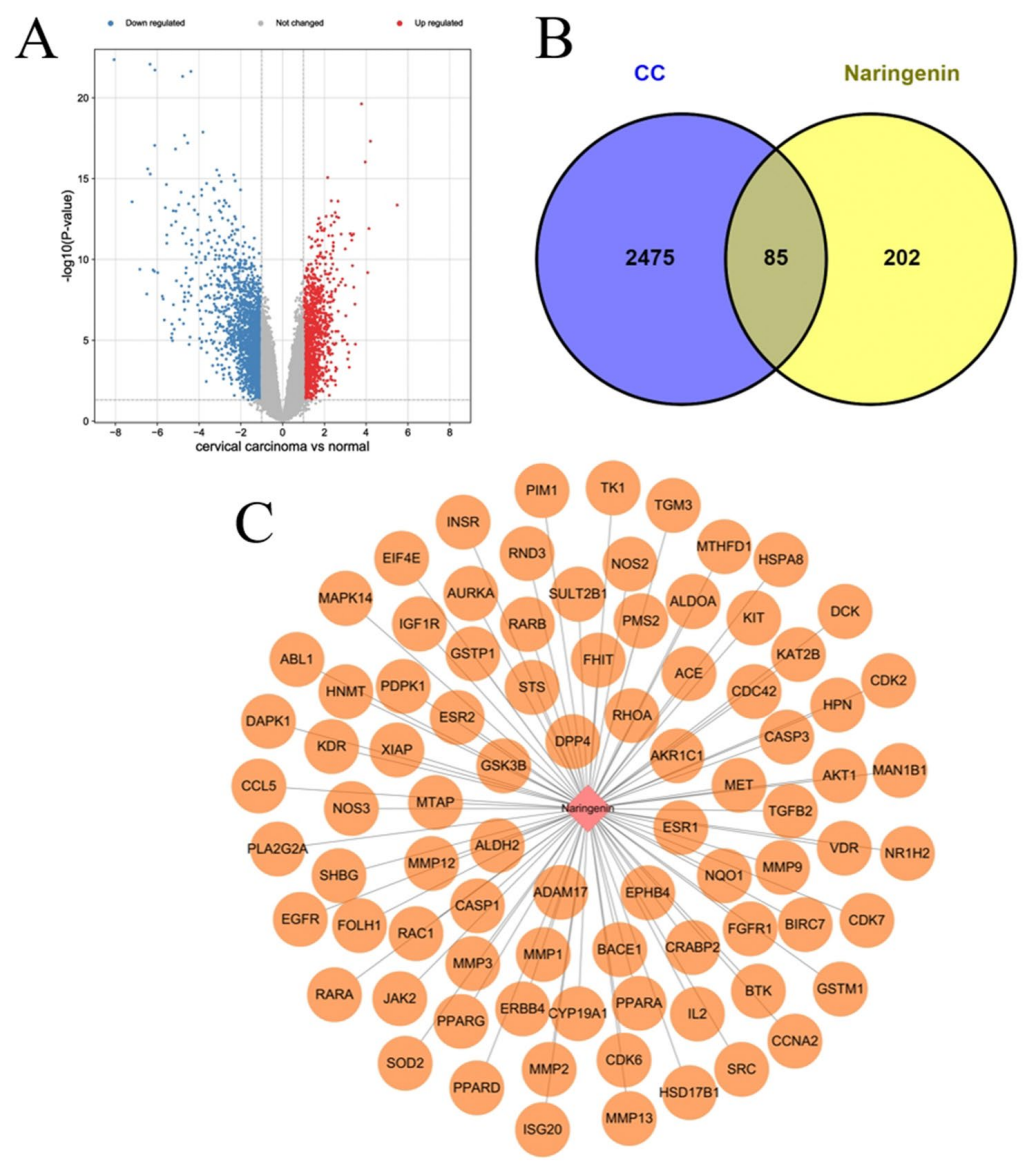
Predictive targets of naringenin and cervical cancer. (**A**) Identification of differentially expressed genes (DEGs) in cervical cancer versus normal cervical tissues. Downregulated genes are represented in blue and upregulated genes in red. (**B**) The Venn diagram illustrating common targets in cervical cancer and naringenin. (**C**) Targets of naringenin relevant to cervical cancer.

We downloaded three sets of cervical cancer data (GSE9750, GSE138080, GSE7803) from the GEO database to procure targets related to cervical cancer, resulting in a collection of 967 differential genes of cervical cancer (Fig. 2A). Further, the Genecards database yielded 1741 related targets. We combined the differential genes from GEO with the disease targets from the Genecards database, removing duplicates, which resulted in 2560 disease targets for cervical cancer (Fig. 2B).

### PPI network construction

**Figure 3:**
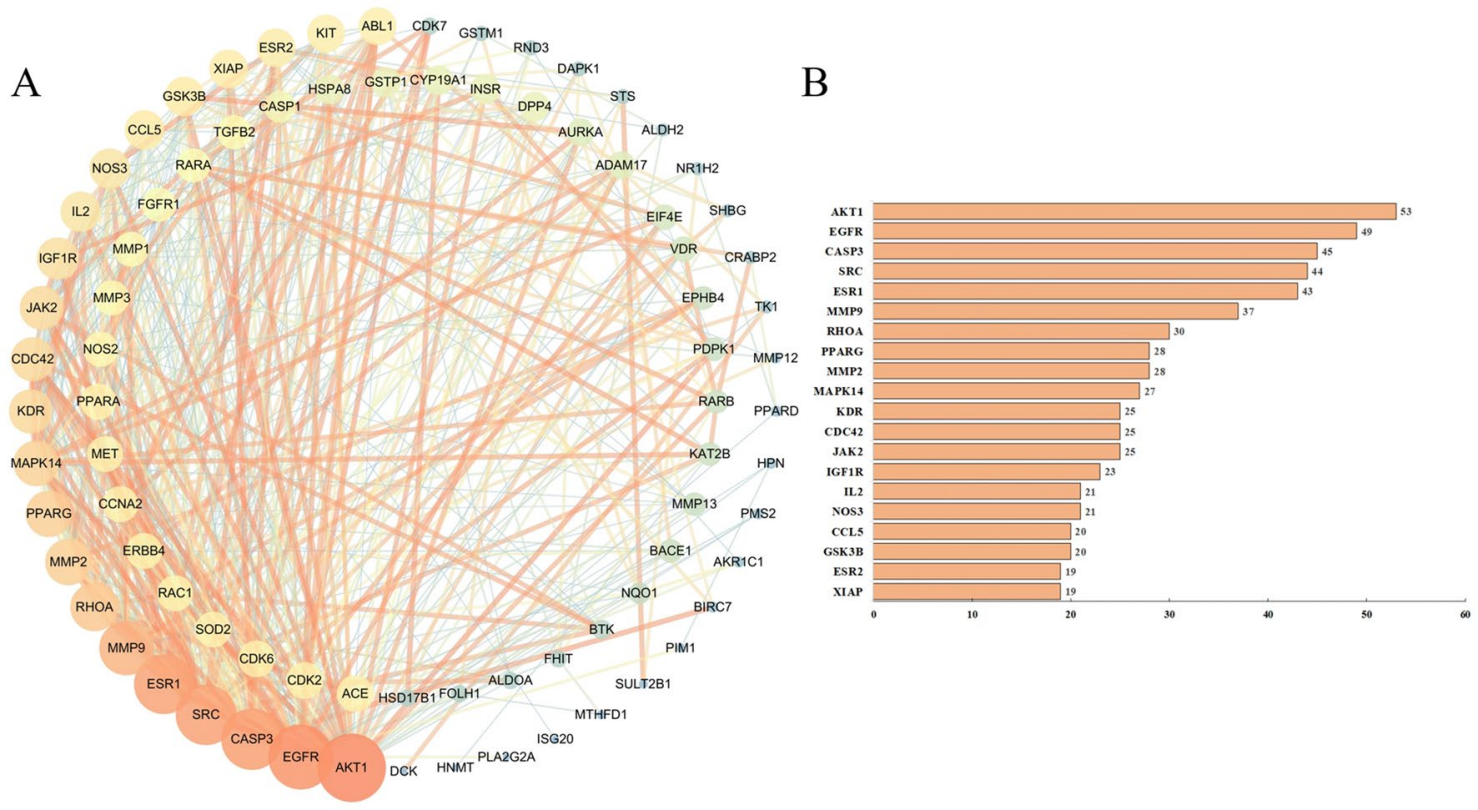
Protein–protein interaction (PPI) network. (**A**) PPI network of naringenin-cervical cancer targets. (**B**) Hub targets of naringenin relevant to cervical cancer.

For a more detailed analysis of potential targets, we extracted 85 common targets from potential targets from naringenin and those relevant to cervical cancer (Fig. 2B, C). To construct a “compound-targets” network, these 85 potential working targets were utilized and analyzed via Cytoscape. The resulting PPI network comprised 577 edges and 82 nodes (Fig. 3A), with the top 20 hub targets including AKT1, EGFR, CASP3, SRC, ESR1, MMP9, MMP2, RHOA, PPARG, MAPK14, among others (Fig. 3B). These results propose these targets as potential naringenin targets for cervical cancer treatment.

### Functional analysis of candidate targets

**Figure 4:**
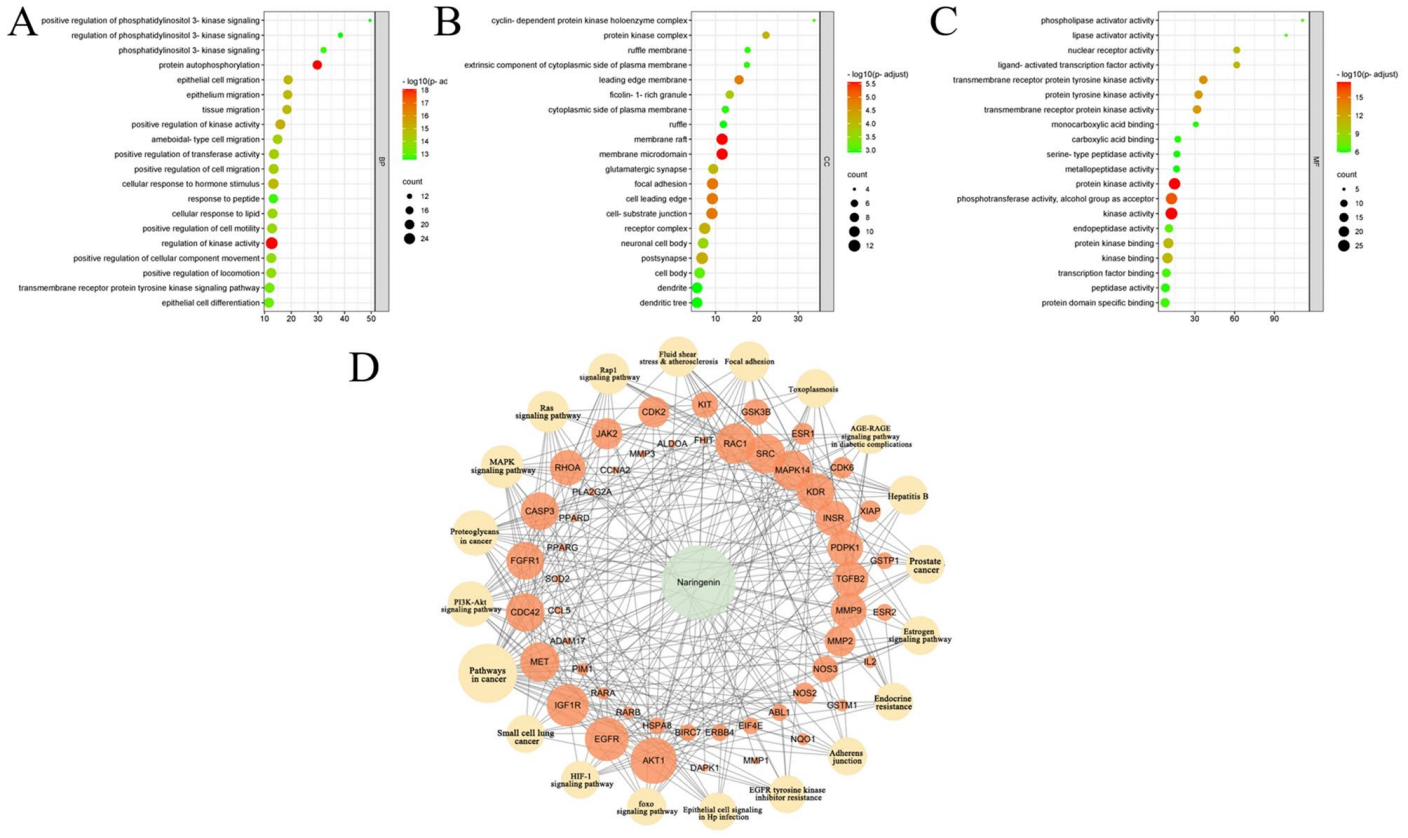
Enrichment analysis of GO and KEGG pathways. (**A**–**C**) Top 20 of GO enrichment analysis for potential targets. (**D**) Top 20 “target-pathways against cervical cancer” network.

To comprehend the biological characteristics of the target gene, we performed a GO function enrichment analysis on the 85 targets, categorizing them into Cell Compounds, Biological Processes and Molecular Functions. Cell Compounds encompassed 67 terms, primarily enriched in membrane raft, membrane microdomain, cell leading edge, focal adhesion, leading edge membrane, cell-substrate junction, receptor complex, protein kinase complex, and more (Fig. 4A). Molecular Functions comprised 105 terms, predominantly enriched in kinase activity, transcription factor binding, nuclear receptor activity, kinase binding, among others (Fig. 4B). Biological Processes covered 1379 terms, mainly focusing on regulation of kinase activity, epithelial cell migration, epithelium migration, epithelial cell differentiation, regulation of 3-kinase signaling, and more (Fig. 4C).

A total of 115 pathways were collected through KEGG enrichment analysis, and the most top 20 signaling pathways were selected for analysis (Fig. 4D). These signaling pathways, including Pathways in cancer (hsa05200), PI3K-Akt pathway (hsa04151), MAPK pathway (hsa04010), Rap1 pathway (hsa04015), AGE-RAGE pathway in diabetic complications (hsa04933), Ras pathway (hsa04014), Foxo pathway (hsa04068), HIF-1 pathway (hsa04066), Estrogen signaling pathway (hsa04915), among others, were related to cancer. These findings suggest that naringenin may play an antagonistic part in cervical cancer via multiple pathways.

### Molecule docking

**Figure 5:**
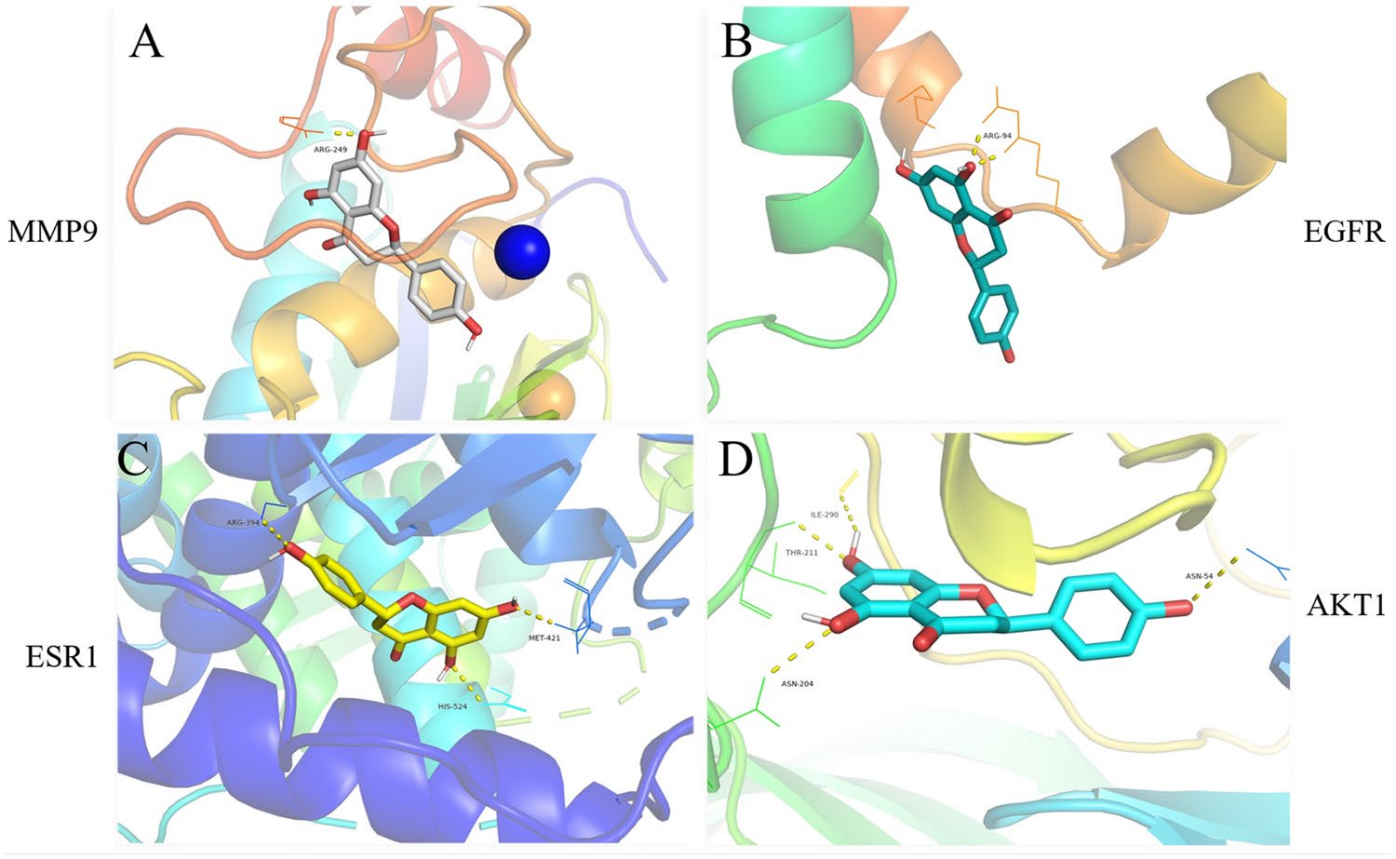
Compound targets subjected to detailed docking simulations with four highest molecular docking affinities (**A**) MMP9, (**B**) EGFR, (**C**) ESR1, (**D**) AKT1.

We utilized Auto Dock Vina for molecular docking to confirm whether the top 10 protein targets interact with naringenin. The results suggested a high binding energy between naringenin and the top 10 targets, particularly with MMP9 (Table 1). As per the molecular docking diagrams, naringenin forms stable hydrogen bonds with amino acid residues ILE-290, THR-211, ASN-204, ASN-54 in the protein structure of AKT1; ARG-94 in the protein structure of EGFR; MET-421, HIS-524, ARG-394 in the protein structure of ESR1; and ARG-249 in the protein structure of MMP9 (Fig. 5). These results suggest that MMP9, EGFR, ESR1, AKT1 could potentially be targets for naringenin.

**Table 1:** Docking scores of naringenin with corresponding potential targets.

Core target	CASP3	EGFR	SRC	MMP9	RHOA
Affinity (kcal/mol)	− 7.7	− 7.2	− 8.8	− 10	− 7.2

Core target	ESR1	MMP2	AKT1	MAPK14	PPARG
Affinity (kcal/mol)	− 8.7	− 9.1	− 9.5	− 8.6	− 7.6

### Impact of naringenin on morphology and viability of Hela cells

**Figure 6:**
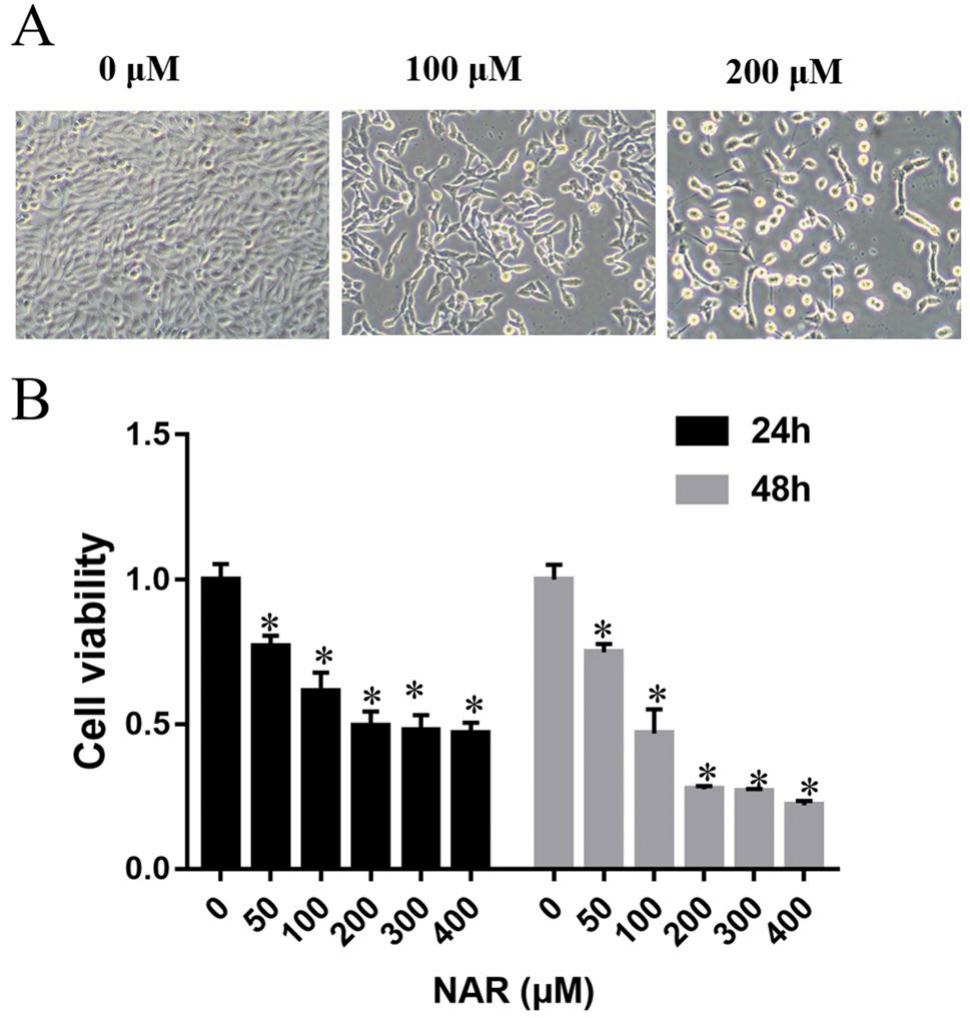
Impact of naringenin on morphology and viability of HeLa cells. (**A**) Microscopic imaging of cell morphology at 200× magnification after incubation with varied concentrations of naringenin for 24 h. (**B**) Viability rates assessed by the CCK-8 assay. Data from three independent experiments are presented as means and standard deviations. *p < 0.05.

To evaluate the influence of naringenin on cervical cancer, we assessed its effect on Hela cell morphology and viability. Following 24 h of incubation with different naringenin concentrations, HeLa cell morphology was recorded under a microscope. Our data indicated that naringenin triggered morphological changes in cells, including shrinkage, rounding, and apoptosis (Fig. 6A). It also diminished HeLa cell viability (Fig. 6B). These results suggest that naringenin may inhibit cellular proliferation in cervical cancer.

### Impact of naringenin on HeLa cell migration and invasion

**Figure 7:**
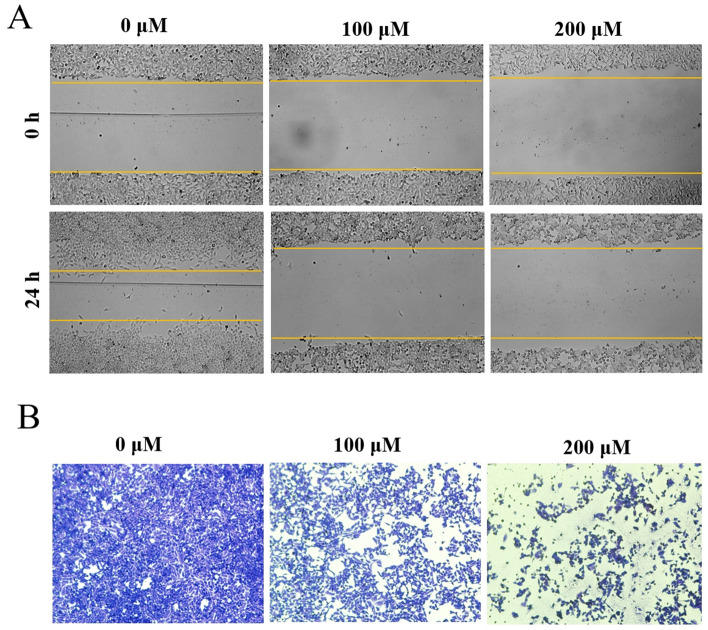
Impact of naringenin on migration and invasion of cervical cancer cells. (**A**) Cell migration assessed through wound healing assay. (**B**) Cell invasion evaluated by Transwell assay. Data are expressed as mean ± SD. *p < 0.05; **p < 0.01.

The wound healing assay was utilized to assess the impact of naringenin on HeLa cells migration. Post 24 h of treatment, the HeLa cells’ migration distance in the control group was significantly less than that in the treatment group (Fig. 7A), suggesting a substantial inhibitory effect of naringenin on HeLa cell migration. Moreover, the Transwell invasion assay indicated that naringenin significantly reduced the number of invading cells in a dose-dependent manner, compared to the control group following pre-incubation with increasing naringenin concentrations for 24 h (Fig. 7B). This evidence suggests that naringenin may inhibit the migration and invasion of cervical cancer cells.

### Naringenin suppression of the EGFR/PI3K/AKT/mTOR pathway

**Figure 8:**
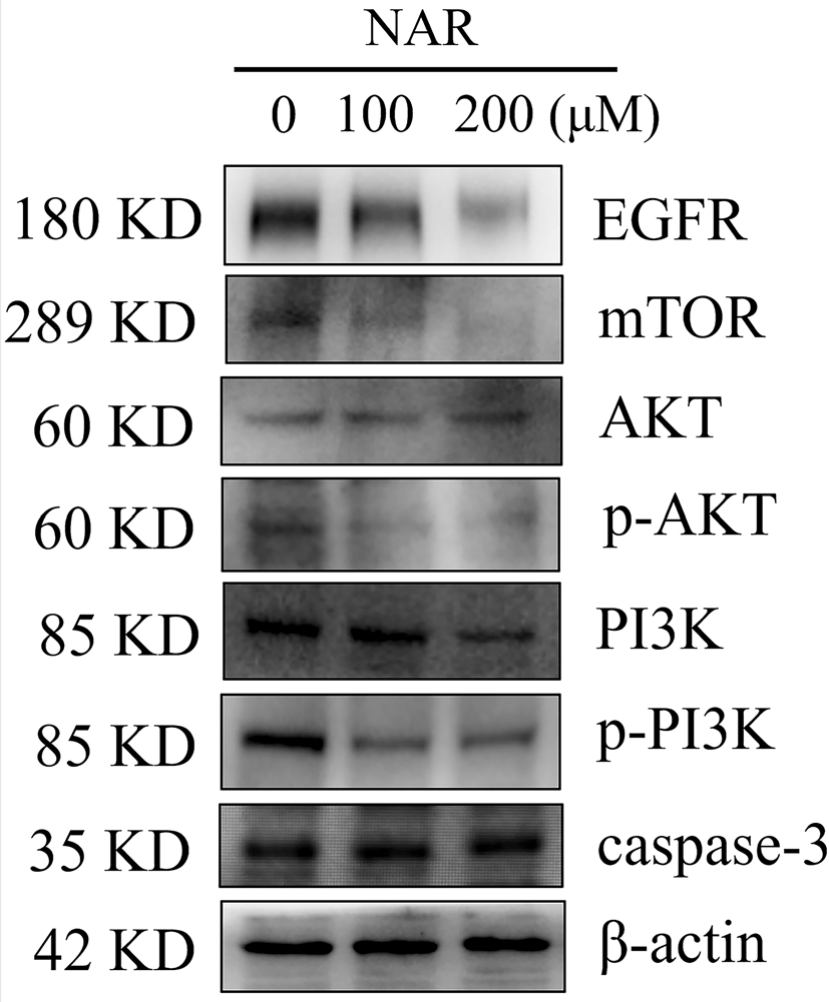
Inhibition of naringenin on EGFR/PI3K/AKT/mTOR pathway. Cropped blots were shown in Fig. 8, and the original blots were presented in Supplementary Fig. 1. Protein expression levels of EGFR, PI3K, p-PI3K, AKT, p-AKT, mTOR, and caspase-3, as detected via western blotting.

To investigate the molecular mechanisms of naringenin in cervical cancer progression, we conducted a study on the protein expression levels of MMP9, EGFR, AKT and caspase-3 in Hela cells exposed to various naringenin concentrations according to our KEGG enrichment analysis and molecular docking results. Additionally, we detected the levels of EGFR downstream proteins, PI3K and mTOR. The results showed that naringenin decreased the expression levels of MMP9 (Supplementary Fig. 1D), EGFR, PI3K, p-PI3K, p-AKT, and mTOR proteins, while enhancing the expression levels of caspase-3 (Fig. 8, original blots were presented in Supplementary Fig. 1). These findings indicate that naringenin can exert an anti-cervical cancer effect by suppressing the EGFR and its downstream PI3K/AKT/mTOR.

## Discussion

Cervical cancer, now recognized as the fourth most prevalent cancer among women, manifests a high incidence and mortality rate^25,26^. Traditional chemotherapy treatments are often marred by issues of drug resistance, diminishing their efficacy over time. There’s a growing interest in Traditional Chinese Medicine (TCM), especially in its use for cancer treatment^27,28^. Derived from natural sources, herbal medicines offer advantages over Western pharmaceuticals, such as greater efficacy and fewer side effects^29,30^, which underscores the pressing need to identify biologically active TCM-derived ingredients for cervical cancer treatment.

Network pharmacology is an emerging discipline rooted in bioinformatics and computer science^31^. This method deviates from the conventional “one drug, one target” strategy, aiming instead to investigate “multiple targets and multiple pathways” inherent in TCM formulas. This approach unravels the relationship between drugs and cancers, and providing a new perspective on drug effects^32^. Many studies suggested that the network pharmacology is a useful tool to uncover the pharmacological mechanisms of TCM^33,34^. For instance, Wu et al., and Zhou et al., employed network pharmacology and experimental validation to elucidate the pharmacological mechanisms of Xihuang Pills in prostate cancer and Sijunzi Decoction in colorectal cancer, respectively^35,36^.

Naringenin, a prominent flavonoid abundant in fruits and Chinese herbal medicines, plays a significant role in disease treatment, particularly in tumorigenesis^37^. Naringenin has been found to induce apoptosis and arrest cell cycles in breast cancer^38^. Additionally, it also reduces the expression of proteins to inhibit proliferation, adhesion, invasion and migration in gastric cancer^39^. Interestingly, recent research also suggests that naringenin possesses inhibitory effects against COVID-19^16^.

In this study, we leveraged network pharmacology and experimental validation to elucidate the mechanism of naringenin in cervical cancer. By accessing the GEO database, we identified 2560 differential genes (DE) from GSE9750, GSE7803, and GSE138080, revealing 85 potential naringenin targets against cervical cancer through PPI and GO and KEGG enrichment analyzes. Several potential targets with cell migration were linked by analysis of GO enrichment, a prediction that we subsequently confirmed using the transwell. Eventually, we selected the top 10 hub targets (AKT1, EGFR, CASP3, SRC, ESR1, MMP9, MMP2, RHOA, PPARG, MAPK14) for molecular docking. All these targets bound to the naringenin molecule, suggesting potential avenues for naringenin-based therapeutics.

The MMP9 gelatinase, a crucial member of the MMP family, contributes significantly to the pathogenesis and progression of numerous cancers. MMP-9 influences the migration and invasion of diverse cancer cells including breast cancer^40^, ovarian cancer^41^, glioblastoma^42^ and liver cancer^43^, among others. Zhang et al.^44^ found that celastrol hampers proliferation, invasion and migration by regulating MMP-9 in HeLa cells, which was consistent with our results. Thus, it is plausible that naringenin could regulate MMP9 to reduce cell invasion and migration.

The pivotal role of caspase-3 is known during apoptosis and tumor growth. Koeppen et al.^45^ revealed that ERBB1 hinders caspase-3 to diminish apoptosis. Lin et al.^46^ illustrated that the enhancement of caspase-3 activation promotes apoptosis in NSCLC. Additionally, another study by Lin et al.^47^ showed that taxol facilitates apoptosis by modulating caspase-3 in nasopharyngeal carcinoma. Previous studies have underscored caspase-3 as an important target for naringenin treatment. For instance, Bao et al.^48^ reported that naringenin inhibits proliferation, migration, invasion, and induces apoptosis through AKT pathway in gastric cancer. Bulzomi et al.^49^ also found that naringenin can activate caspase-3 in breast cancer. These findings align with our results.

Epidermal growth factor receptor (EGFR) possesses tyrosine kinase activity and is frequently overexpressed in tumors^50^. EGFR can activate downstream signaling pathways, thereby promoting tumor cell proliferation, migration, and invasion through ligand binding^51,52^. Previous studies have shown that EGFR is often overexpressed and associated with migration and invasion in cervical cancer^53^. Our study demonstrates that naringenin effectively inhibits the expression of the EGFR-mediated PI3K/AKT/mTOR signaling pathway, which is consistent with previous findings. The PI3K/AKT/mTOR is an important pathway downstream of EGFR signalling^54^. The PI3K/AKT/mTOR signalling is known to play an essential part in proliferation, migration, and invasion of cancer cells and presents a potential therapeutic biomarker^55^. Previous studies demonstrated that PI3K/AKT/mTOR pathway is a vital way to treat diseases in traditional Chinese medicine. For instance, Yang et al.^56^ reported that PI3K/AKT /mTOR pathway is a significant target for natural medicines on TNBC. Farhanet al.^57^ discovered that Artemisinin regulated migration and invasion of uveal melanoma through PI3K/AKT/mTOR pathway. Our study found PI3K/AKT pathway to be the most significantly enriched pathway, and the subsequent WB experiment demonstrated that naringenin suppresses the activation of PI3K/AKT/mTOR pathway. Thus, this study shows that naringenin could inhibit proliferation, migration, and invasion of HeLa cells through EGFR/PI3K/AKT/mTOR pathway.

In summary, we demonstrated the potential molecular mechanism of naringenin in treating cervical cancer through network pharmacology and molecular docking, providing fresh insights into its mechanism against cervical cancer. Our integrated analysis of databases revealed that MMP-9, EGFR, AKT, and caspase-3 may be the drug targets of naringenin in cervical cancer. Our cellular experiments showed that naringenin can inhibit the proliferation, migration, and invasion of cervical cancer cells, and further western blot confirmed that naringenin achieves these effects by EGFR/PI3K/AKT/mTOR pathway. Additionally, we also discovered that naringenin can modulate other signaling pathways to exert anti-cancer effects, and these other pathways warrant further investigation. In order to further explore the potential clinical utility of naringenin, future studies should include more extensive cellular experiments and in vivo animal studies.

## Conclusion

**Figure 9:**
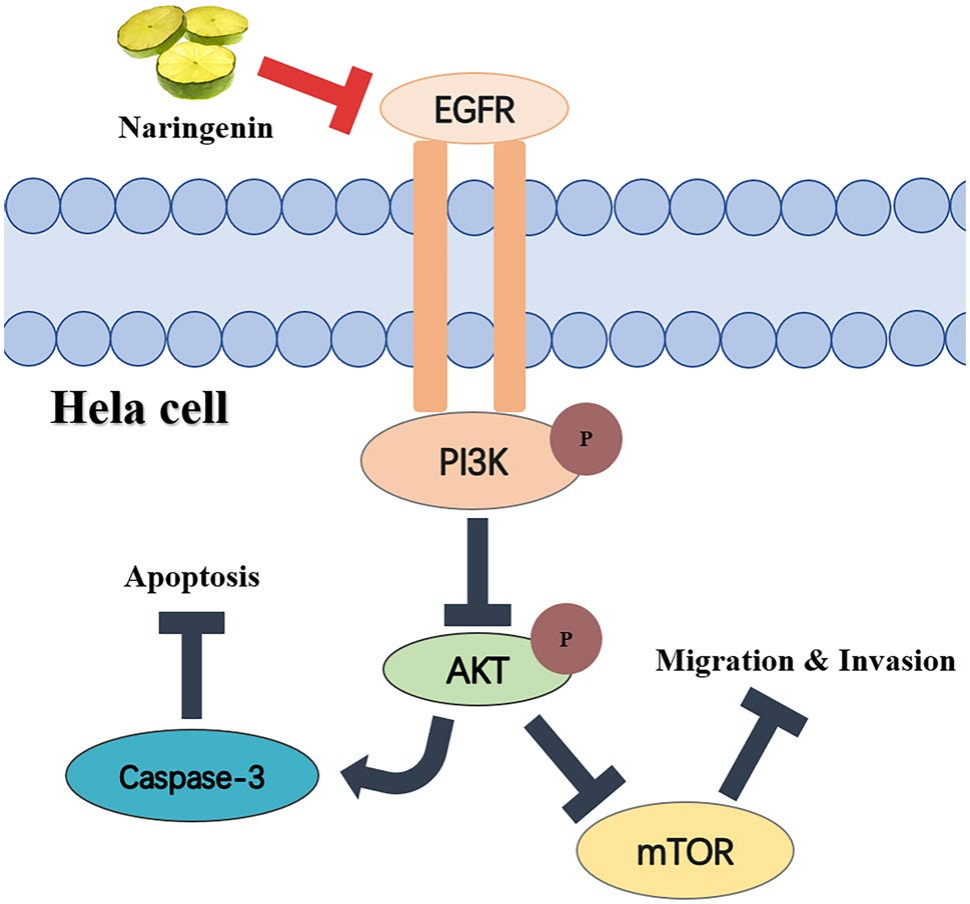
Schematic diagram of naringenin in anti-cervical cancer.

In this study, through network pharmacology and experimental evidence, we have demonstrated that naringenin exerts therapeutic effects on cervical cancer via multiple mechanisms, including the inhibition of proliferation, migration and invasion through the regulation of the EGFR/PI3K/AKT/mTOR pathway. The overview of this study is shown in Fig. 9. This study introduces a novel approach to understanding the effects of naringenin on cervical cancer, providing theoretical support for new drugs for the treatment of cervical cancer.

### Supplementary Information

Supplementary Figure 1.

Supplementary Information.

## Data Availability

All supporting data are included within the article, and all the data generated in this article are available from the first author on reasonable request.
