# Supplementary material for: Network pharmacology combined with experimental verification to explore the potential mechanism of naringenin in the treatment of cervical cancer
Source: Sci Rep. 2024 Jan 22;14:1860. doi: 10.1038/s41598-024-52413-9 (PMC10803340; doi:10.1038/s41598-024-52413-9)
Supplement: Supplementary file 1 — Supplementary Figure 1. [file 41598_2024_52413_MOESM1_ESM.pptx]

## Slide 1
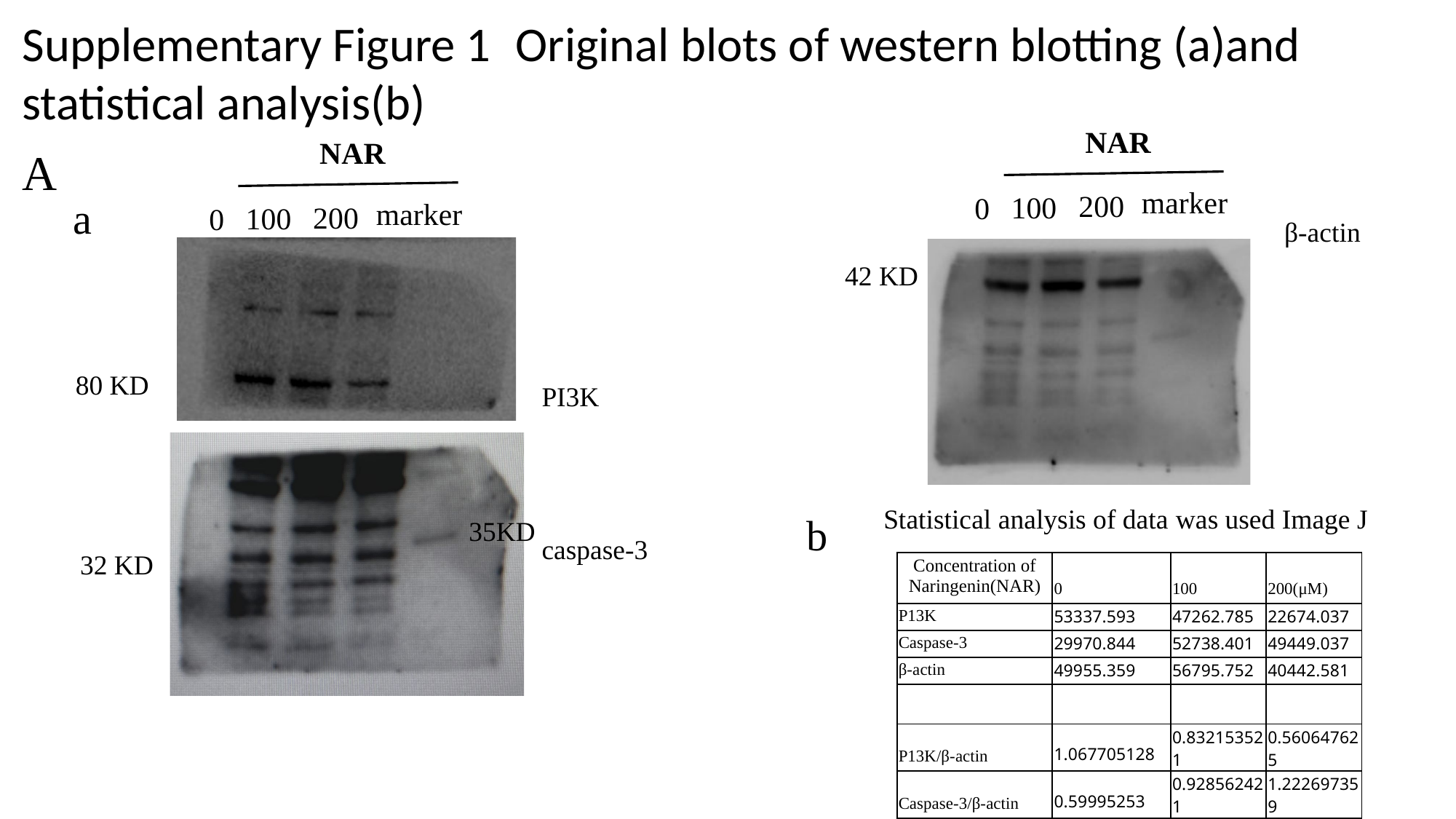

Supplementary Figure 1 Original blots of western blotting (a)and statistical analysis(b)
NAR
NAR
A
marker
200
100
0
a
marker
200
100
0
β-actin
 42 KD
 80 KD
PI3K
 b
Statistical analysis of data was used Image J
35KD
caspase-3
 32 KD
| Concentration of Naringenin(NAR) | 0 | 100 | 200(μM) |
| --- | --- | --- | --- |
| P13K | 53337.593 | 47262.785 | 22674.037 |
| Caspase-3 | 29970.844 | 52738.401 | 49449.037 |
| β-actin | 49955.359 | 56795.752 | 40442.581 |
| | | | |
| P13K/β-actin | 1.067705128 | 0.832153521 | 0.560647625 |
| Caspase-3/β-actin | 0.59995253 | 0.928562421 | 1.222697359 |

## Slide 2
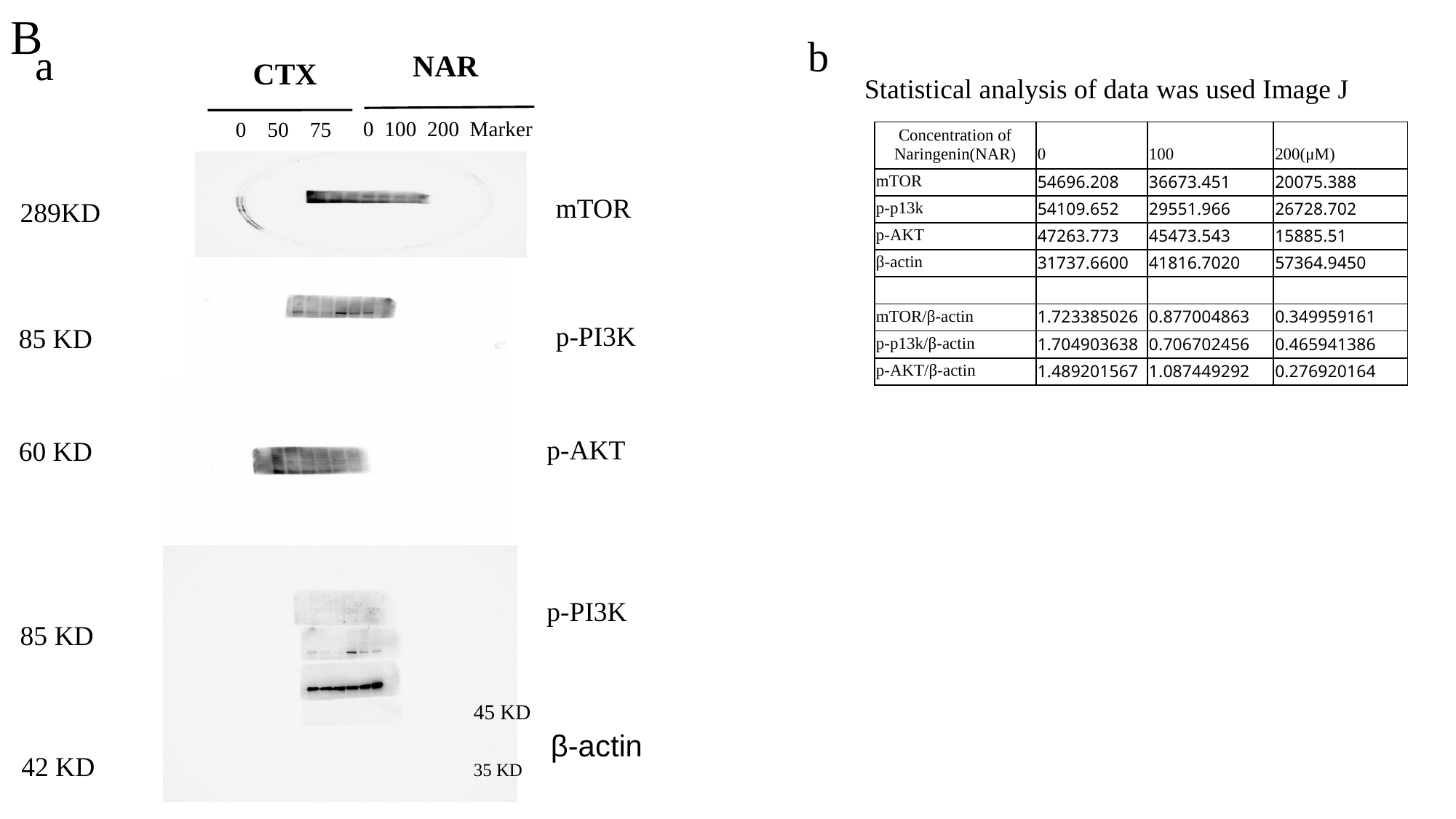

B
b
a
NAR
CTX
Statistical analysis of data was used Image J
0 100 200 Marker
0 50 75
| Concentration of Naringenin(NAR) | 0 | 100 | 200(μM) |
| --- | --- | --- | --- |
| mTOR | 54696.208 | 36673.451 | 20075.388 |
| p-p13k | 54109.652 | 29551.966 | 26728.702 |
| p-AKT | 47263.773 | 45473.543 | 15885.51 |
| β-actin | 31737.6600 | 41816.7020 | 57364.9450 |
| | | | |
| mTOR/β-actin | 1.723385026 | 0.877004863 | 0.349959161 |
| p-p13k/β-actin | 1.704903638 | 0.706702456 | 0.465941386 |
| p-AKT/β-actin | 1.489201567 | 1.087449292 | 0.276920164 |
mTOR
289KD
p-PI3K
85 KD
p-AKT
60 KD
p-PI3K
85 KD
45 KD
β-actin
42 KD
35 KD

## Slide 3
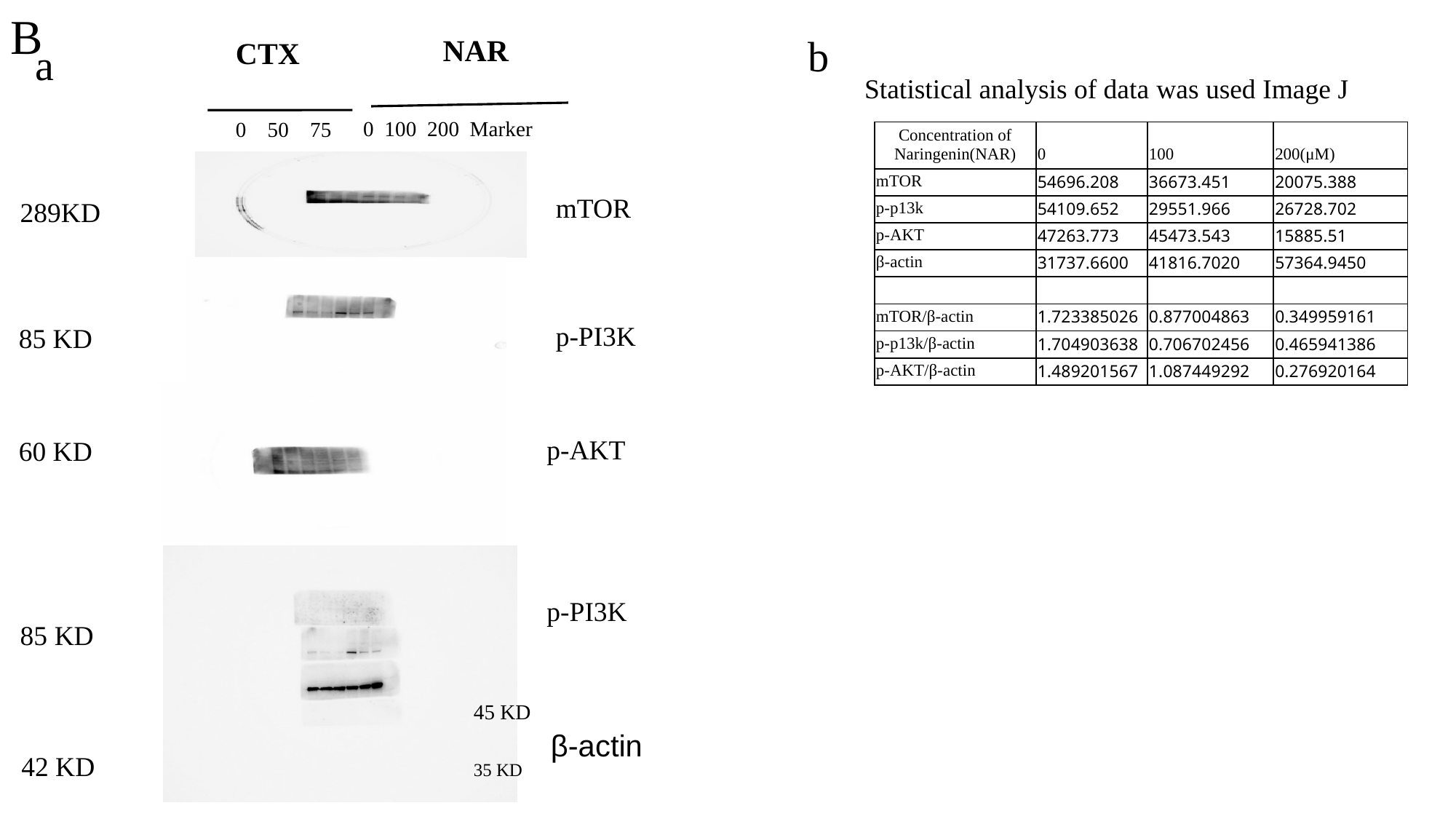

B
b
NAR
CTX
a
Statistical analysis of data was used Image J
0 100 200 Marker
0 50 75
| Concentration of Naringenin(NAR) | 0 | 100 | 200(μM) |
| --- | --- | --- | --- |
| mTOR | 54696.208 | 36673.451 | 20075.388 |
| p-p13k | 54109.652 | 29551.966 | 26728.702 |
| p-AKT | 47263.773 | 45473.543 | 15885.51 |
| β-actin | 31737.6600 | 41816.7020 | 57364.9450 |
| | | | |
| mTOR/β-actin | 1.723385026 | 0.877004863 | 0.349959161 |
| p-p13k/β-actin | 1.704903638 | 0.706702456 | 0.465941386 |
| p-AKT/β-actin | 1.489201567 | 1.087449292 | 0.276920164 |
mTOR
289KD
p-PI3K
85 KD
p-AKT
60 KD
p-PI3K
85 KD
45 KD
β-actin
42 KD
35 KD

## Slide 4
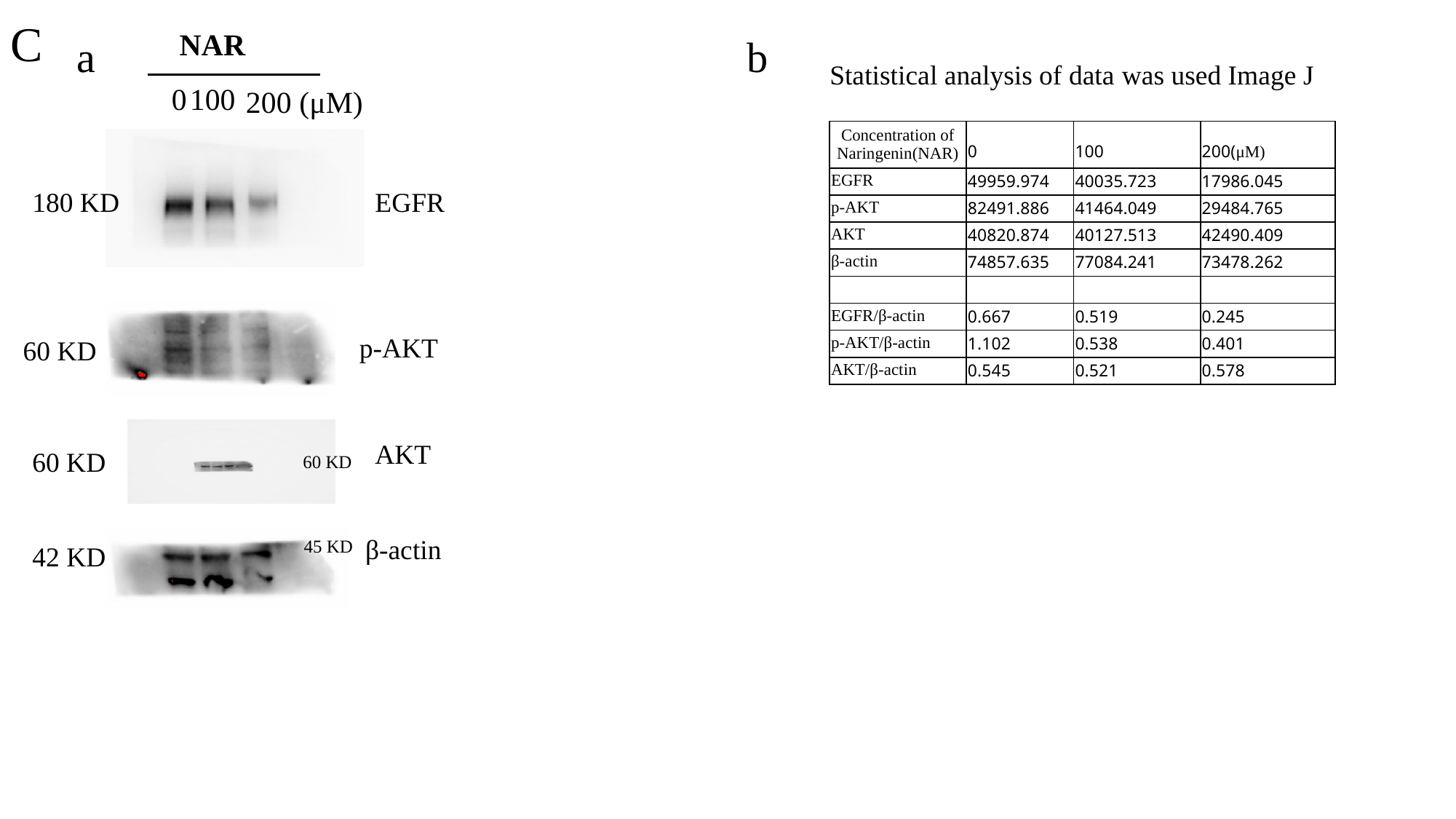

C
NAR
a
b
Statistical analysis of data was used Image J
0
100
200 (μM)
| Concentration of Naringenin(NAR) | 0 | 100 | 200(μM) |
| --- | --- | --- | --- |
| EGFR | 49959.974 | 40035.723 | 17986.045 |
| p-AKT | 82491.886 | 41464.049 | 29484.765 |
| AKT | 40820.874 | 40127.513 | 42490.409 |
| β-actin | 74857.635 | 77084.241 | 73478.262 |
| | | | |
| EGFR/β-actin | 0.667 | 0.519 | 0.245 |
| p-AKT/β-actin | 1.102 | 0.538 | 0.401 |
| AKT/β-actin | 0.545 | 0.521 | 0.578 |
180 KD
EGFR
p-AKT
60 KD
AKT
60 KD
60 KD
45 KD
β-actin
42 KD

## Slide 5
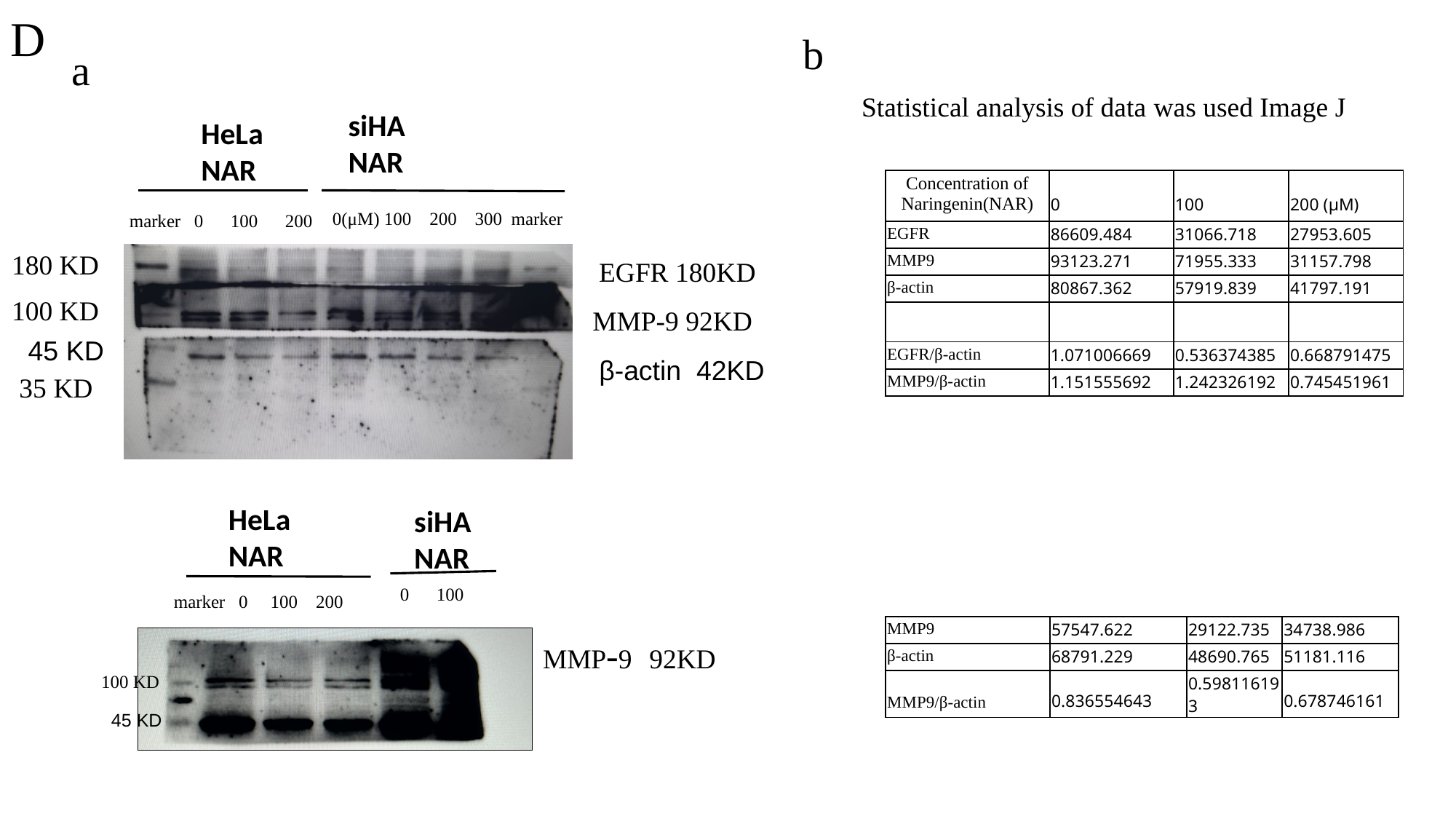

D
b
a
Statistical analysis of data was used Image J
siHANAR
HeLaNAR
| Concentration of Naringenin(NAR) | 0 | 100 | 200 (μM) |
| --- | --- | --- | --- |
| EGFR | 86609.484 | 31066.718 | 27953.605 |
| MMP9 | 93123.271 | 71955.333 | 31157.798 |
| β-actin | 80867.362 | 57919.839 | 41797.191 |
| | | | |
| EGFR/β-actin | 1.071006669 | 0.536374385 | 0.668791475 |
| MMP9/β-actin | 1.151555692 | 1.242326192 | 0.745451961 |
0(μM) 100 200 300 marker
marker 0 100 200
180 KD
EGFR 180KD
100 KD
MMP-9 92KD
45 KD
β-actin 42KD
35 KD
HeLaNAR
siHANAR
 0 100
marker 0 100 200
| MMP9 | 57547.622 | 29122.735 | 34738.986 |
| --- | --- | --- | --- |
| β-actin | 68791.229 | 48690.765 | 51181.116 |
| MMP9/β-actin | 0.836554643 | 0.598116193 | 0.678746161 |
MMP-9 92KD
100 KD
45 KD

## Slide 6
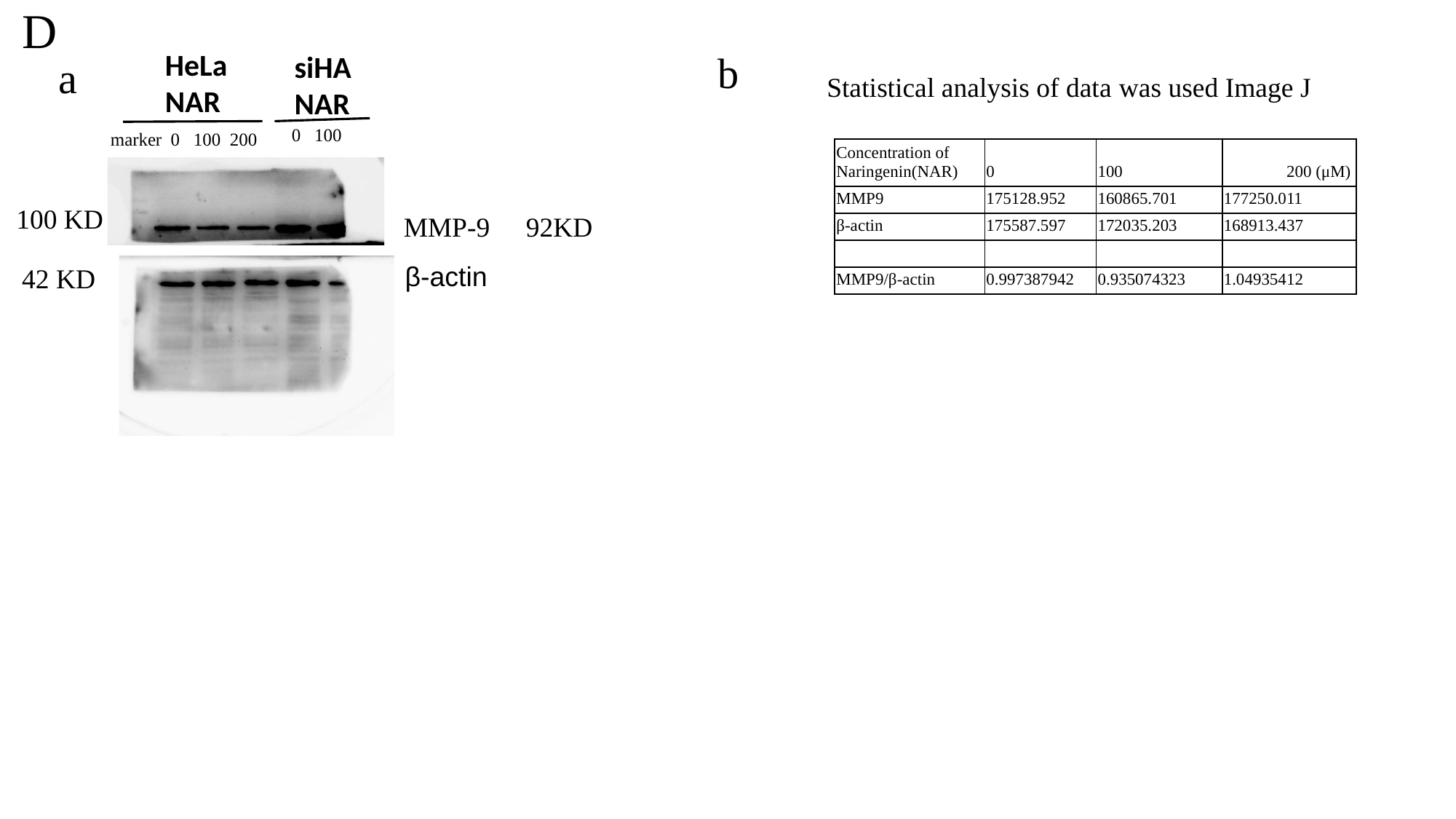

D
HeLaNAR
b
siHANAR
a
Statistical analysis of data was used Image J
 0 100
marker 0 100 200
| Concentration of Naringenin(NAR) | 0 | 100 | 200 (μM) |
| --- | --- | --- | --- |
| MMP9 | 175128.952 | 160865.701 | 177250.011 |
| β-actin | 175587.597 | 172035.203 | 168913.437 |
| | | | |
| MMP9/β-actin | 0.997387942 | 0.935074323 | 1.04935412 |
100 KD
MMP-9
 92KD
β-actin
42 KD

## Slide 7
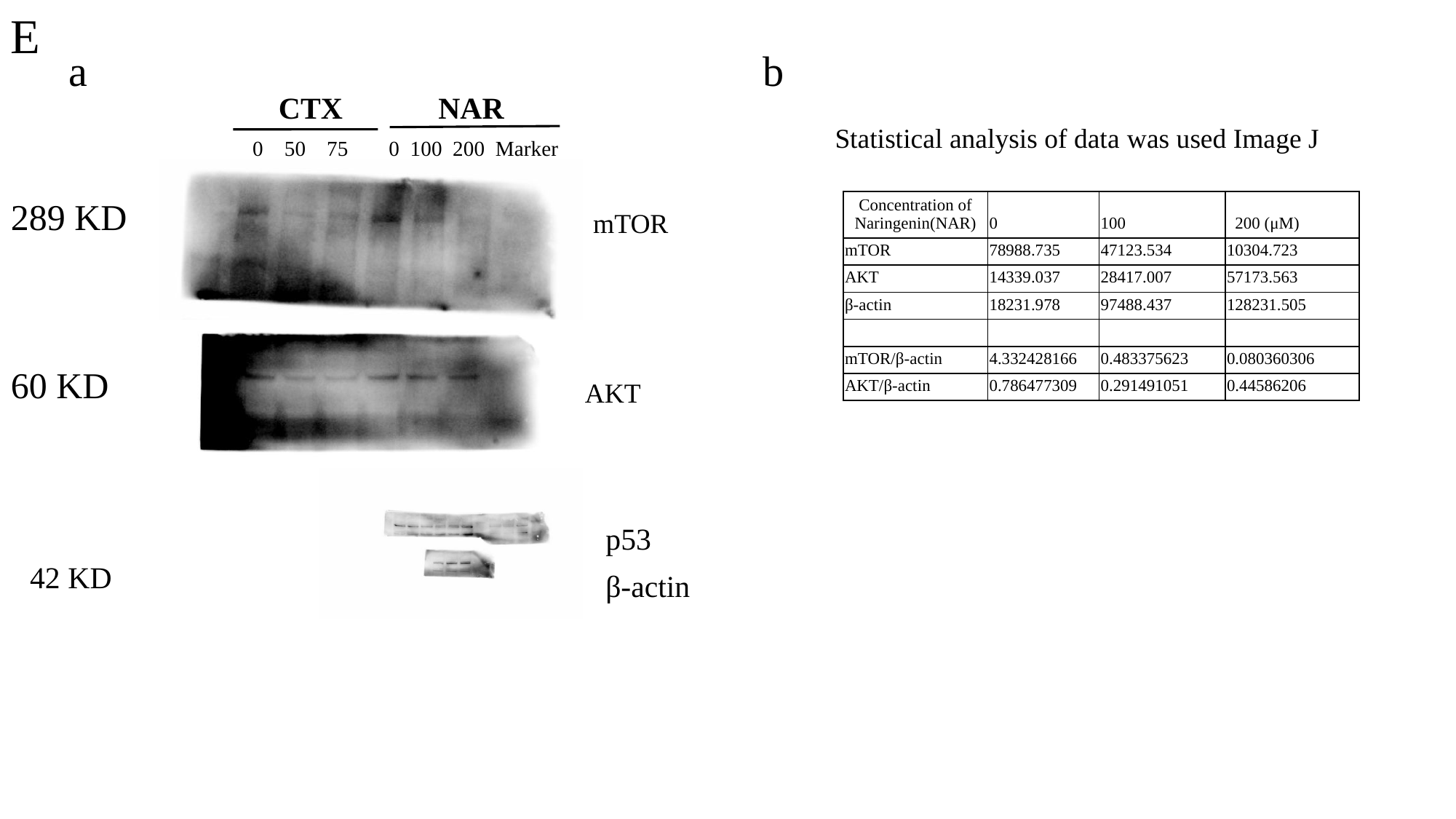

E
a
b
CTX
NAR
Statistical analysis of data was used Image J
0 50 75
0 100 200 Marker
289 KD
mTOR
| Concentration of Naringenin(NAR) | 0 | 100 | 200 (μM) |
| --- | --- | --- | --- |
| mTOR | 78988.735 | 47123.534 | 10304.723 |
| AKT | 14339.037 | 28417.007 | 57173.563 |
| β-actin | 18231.978 | 97488.437 | 128231.505 |
| | | | |
| mTOR/β-actin | 4.332428166 | 0.483375623 | 0.080360306 |
| AKT/β-actin | 0.786477309 | 0.291491051 | 0.44586206 |
60 KD
AKT
p53
 42 KD
β-actin
